# Persistence and Complex Evolution of Fluoroquinolone-Resistant *Streptococcus pneumoniae* Clone

**DOI:** 10.3201/eid2005.130142

**Published:** 2014-05

**Authors:** Debby Ben-David, Mitchell J. Schwaber, Amos Adler, Samira Masarwa, Rotem Edgar, Shiri Navon-Venezia, David Schwartz, Nurith Porat, Tali Kotlovsky, Nikolay Polivkin, Irina Weinberg, Avraham Lazary, Nissim Ohana, Ron Dagan

**Affiliations:** National Center for Infection Control, Tel Aviv, Israel (D. Ben-David, M.J. Schwaber, A. Adler, S. Masarwa, R. Edgar, S. Navon-Venezia);; Sourasky Medical Center, Tel Aviv (D. Schwartz, N. Porat, T. Kotlovsky, R. Dagan);; Ben-Gurion University of the Negev, Beer-Sheva (N. Porat, R. Dagan);; Reuth Medical Center, Tel Aviv (N. Polivkin, I. Weinberg, A. Lazary, N. Ohana)

**Keywords:** *Streptococcus pneumoniae*, fluoroquinolones, antimicrobial drug resistance, long-term acute care hospital, clonal evolution, bacteria

## Abstract

Prolonged outbreaks of multidrug-resistant *Streptococcus pneumoniae* in health care facilities are uncommon. We found persistent transmission of a fluroquinolone-resistant *S. pneumoniae* clone during 2006–2011 in a post–acute care facility in Israel, despite mandatory vaccination and fluoroquinolone restriction. Capsular switch and multiple antimicrobial nonsusceptibility mutations occurred within this single clone. The persistent transmission of fluoroquinolone-resistant *S. pneumoniae* during a 5-year period underscores the importance of long-term care facilities as potential reservoirs of multidrug-resistant streptococci.

## Abstract 2

This clone has persisted in a post–acute care facility for >5 years.

Institutionalized persons, particularly those >65 years of age, are at high risk for pneumococcal infections (*1*–*3*). The rate of sporadic pneumococcal diseases for nursing home residents is almost 20 times higher than for elderly persons living in the community (*1*).

In September 2008 in Israel, after report of an invasive pneumococcal disease caused by a fluoroquinolone-resistant *Streptococcus pneumoniae* (FQRSP) strain in a patient who had been transferred from a post–acute care facility, an investigation led to discovery that this phenotype had been endemic in the facility for at least 2 years. In the index case-patient, an 81-year-old woman with dementia, bilateral pneumonia and acute respiratory failure developed while she was in a post-acute care facility. Because her condition rapidly deteriorated, she was transferred to a tertiary acute care facility and died within 48 hours. Blood cultures recovered FQRSP. Because fluoroquinolone resistance among *S. pneumoniae* strains is rare in Israel and was infrequently reported in previous pneumococcal outbreaks worldwide (*4*,*5*), we conducted an investigation and attempted to implement measures to limit the spread of the resistant strains. Here we describe prolonged endemicity of a FQRSP clone in the post–acute care facility, its molecular epidemiology, and the effect of infection control measures implemented. The analysis and reporting were approved by the jurisdictional institutional review board of Sourasky Medical Center (Tel Aviv, Israel).

## Methods

### Setting

The facility is a 307-bed, post–acute care hospital (273 adults, 34 children). Patients are grouped into the following wards: adults and children on long-term mechanical ventilation, rehabilitation, and skilled nursing care. Median duration of hospitalization is 48 days in the rehabilitation wards, 152 days in the skilled nursing wards, and 313 days in the long-term mechanical ventilation wards.

### Outbreak Investigation

In September 2008, after report of the patient with FQRSP bloodstream infection, we reviewed the clinical microbiology database at Sourasky Medical Center from January 2006 onward. A clinical case was defined as FQNSP isolated from a clinical specimen from any source. Fifty-two clinical cases of FQRSP were identified, and an outbreak investigation was initiated. However, medical records were found only for 43 patients, and clinical documentation of physical examination and clinical assessment findings was sparse. We reviewed the medical records for clinical and epidemiologic data, including patients’ demographics, underlying diseases, and antimicrobial drug exposure, in the 3 months preceding the isolation.

### Interventions

The first intervention, vaccination, began in November 2008. Vaccination with 23-valent pneumococcal polysaccharide vaccine (PPV23) was made mandatory for all patients >2 years of age who were admitted to the facility. In addition, all adult patients not previously vaccinated with PPV23 received 1 dose during November and December 2008. Hospitalized and newly admitted unvaccinated children <5 years of age received 1 dose of 7-valent pneumococcal polysaccharide vaccine (PPV7). In addition, children 2–4 years of age received 1 dose of PPV23.

The second intervention, fluoroquinolone restriction, was implemented from January 2010 through October 2011 in all wards. Under the restriction policy, fluoroquinolones were prescribed only after approval by a designated staff physician. Fluoroquinolones were not used for empirical therapy and were approved for definitive therapy only when other therapeutic options were unavailable.

Total fluoroquinolone use since January 2009 as recorded by the central pharmacy was aggregated for each ward into defined daily doses (DDDs) per 1,000 bed-days, as recommended by the World Health Organization (*6*). The study was divided into 3 periods: baseline period (January 2006–January 2009); phase 1: post–vaccination period (February 2009–December 2009); and phase 2: fluoroquinolone-restriction period (January 2010–October 2011).

### Point-Prevalence Surveillances

To determine the extent of FQNSP spread among patients and staff members, point-prevalence surveillance was conducted during the baseline period in January 2009. A convenience sample of ≈10 patients from each of the 9 wards and 20 staff members from these wards were selected for screening. Oropharyngeal and nasopharyngeal swab samples were taken from all persons by using the Transwab Pernasal Amies Plain wire swabs (Medical Wire, Corsham, UK). Endotracheal aspirates were obtained by using a suction catheter introduced through tracheostomy tubes.

To evaluate the effects of vaccination and fluoroquinolone restriction on FQNSP, we conducted follow-up point-prevalence surveillances during December 2009–January 2010 and May–June 2011. In the second and third surveys we increased the sample size, screening all patients hospitalized in a convenience sample of 3 wards in addition to a sample of 10 patients from each of the other wards. These wards represented all types of wards in the facility.

### Microbiological Methods

#### Pneumococcal Isolation, Identification, and Susceptibility Testing

Specimens were transported to the clinical laboratory at Sourasky Medical Center. Specimens were streaked onto either tryptic soy agar with 5% sheep blood and gentamicin (5 mg/L) (first and second surveys) or Streptococcal Select Agar plates (Hy-labs, Rehovot, Israel) (third survey) and incubated overnight at 37°C in 5% CO_2_; the 2 methods were validated in our laboratory. The selective plates were compared with tryptic soy agar–5% sheep blood and had similar ability to support the growth of *S. pneumoniae* (data not shown). Pneumococcal identification and antimicrobial susceptibility testing were performed with the VITEK-2 system by using the GP and AST-GP68 cards, respectively (bioMérieux, Marcy l’Etoile, France) according to Clinical and Laboratory Standards Institute guidelines (*7*). MICs of ofloxacin, levofloxacin, and moxifloxacin also were tested in representative isolates by Etest (AB Biodisk, Solna, Sweden). Pneumococci were defined as resistant to ofloxacin if the ofloxacin MIC was >8 μg/mL (FQRSP). Fluoroquinolone intermediately resistant *S. pneumoniae* (FQISP) was defined as MIC = 4 μg/mL. Penicillin-nonsusceptible strains were defined as those with penicillin MIC >2 μg/mL.

#### Clonal Analysis

Serogrouping and serotyping were performed by the quellung reaction using antiserum provided by Statens Serum Institute (Copenhagen, Denmark) (*8*). We determined the genetic relatedness of *S*. *pneumoniae* strains by pulsed-field gel electrophoresis (PFGE) analysis, as described (*9*). Selected isolates representing all PFGE clusters were characterized by multilocus sequence typing (MLST) as described by Enright and Spratt (*10*). The sequences (alleles) at each locus were compared with those at the MLST website (www.mlst.net), and sequence types (STs) were assigned.

#### Mechanisms of Fluoroquinolone Resistance

Mutations in the quinolone resistance–determining regions (QRDR) of genes encoding subunits of topoisomerase IV and DNA gyrase were assessed. Representatives from each serotype in PFGE and resistance level were tested. Primers amplifying the QRDR (*11*) were designed for each of the 4 genes: *parC* primers, F-CAAAACATGTCCCTGGAGGA and R-GCAGCATCTATGACCTCAGC; *parE* primers, F-TCAAGTCTGCCATTACCAAGG and R-ACCCGCACCAATGGTATAAA; *gyrA* primers, F2-GACAAAGGAGATGAAGGCAAG and R2-GAAAATCTGGTCCAGGCAAG; *gyrB* primers, F-GGGAAATAGCGAAGTGGTCA and R-GTACGAATGTGGGCTCCAT. PCR on lysates with primers as above using Hot Star Taq (QIAGEN, Hilden, Germany) was performed as follows: 95°C for 15 min and 39 cycles of 94°C for 1 min, 56°C for 1 min, 72°C for 1 min, followed by an extension step of 72°C for 10 min, and the products were sequenced (HyLab, Rehovot, Israel). Sequences were analyzed by BLAST (http://blast.ncbi.nlm.nih.gov) against 1 of the 2 identical sequenced pneumococcal strains in the database (NC_008533 *Streptococcus pneumoniae* D39 and AE007317).

### Statistical Analysis

The effect of the intervention was assessed during the 3 periods: baseline period, phase 1 (February 2009–December 2009), and phase 2 (January 2010–October 2011). We assumed a lag time of 2–4 weeks for demonstrating vaccine efficacy and therefore defined the postvaccination period as beginning ≈1 month after vaccination. We used segmented Poisson regression analysis of interrupted time series to compare FQRSP incidence during the 3 periods. FQRSP incidence was measured as cases per 10,000 patient-days. A p value <0.05 was considered significant.

## Results

### Clinical Cases of FQRSP during the Baseline Period

Before November 2008, pneumococcal vaccine was not administered at admission to the facility. Only 40 (13%) of 310 patients had received pneumococcal vaccine before hospitalization. During January 2006–December 2008, *S. pneumoniae* was isolated from 66 patients. Of these, 52 (79%) isolates were fluoroquinolone-resistant, 11 (17%) were fluoroquinolone-susceptible, and 3 (5%) were intermediate. FQRSP was isolated predominantly from sputum (51 of 52 isolates). Most (45 [87%] of 52) FQRSP isolates were nonsusceptible to penicillin. Resistance to erythromycin, tetracycline, and trimethoprim/sulfamethoxazole was found in 8%, 6%, and 13% of isolates, respectively. In contrast to the high FQRSP isolation rate in the facility, the same laboratory detected fluoroquinolone resistance in only 4 (2%) of 240 of pneumococcal isolates from sputum cultures from patients in a tertiary acute care facility during that period.

Medical records were available for 43 patients with FQRSP. Their median age was 59 years (range 20–94 years). Twenty-nine (67%) patients were male. The median length of stay at the facility before FQRSP isolation was 329 days (range 3 days–26 years). Antimicrobial drug use was high in this population: 51% of patients had received >1 antimicrobial agents in the 3 months preceding isolation. Only 5 (12%) of the 43 patients received fluoroquinolones in the 3 months preceding isolation. Symptoms associated with detection of FQRSP included fever (23 [53%] patients) and respiratory deterioration (27 [63%]).

### Baseline Point-Prevalence Surveillance: Serotyping and Clonal Analysis

The baseline survey comprised 84 (93%) of the 90 eligible patients and 20 (4%) of 525 health care workers. Asymptomatic colonization with *S. pneumoniae* was detected among 20 patients (16 [23%] of 69 adults; 4 [27%] of 15 children) and 1 (5%) health care worker. Of the colonized patients, 12 (60%) had FQNSP. This represented 14% of the sampled patients: 10 (14%) of 69 adults, all with FQRSP; and 2 (13%) of 15 children, all with FQISP. The isolate from the health care worker was fluoroquinolone susceptible.

**Figure 1:**
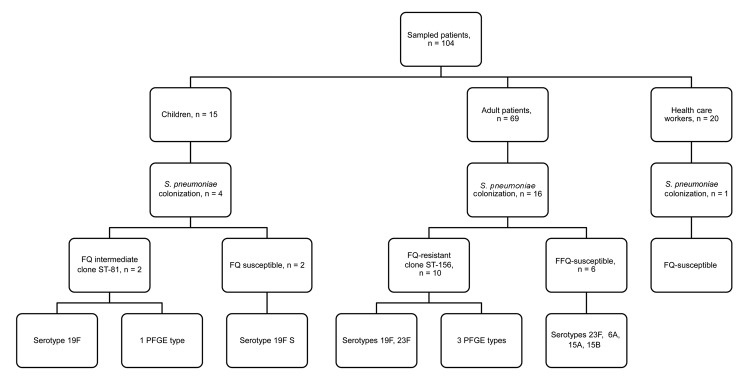
Serotyping and clonal analysis of the first point-prevalence survey of *Streptococcus pneumoniae* infection in a post–acute care facility, Israel, 2006–2011. FQ fluoroquinolone; ST, sequence type; FQISP, fluoroquinolone-intermediate *S. pneumoniae*; FQRSP fluoroquinolone-resistant *S. pneumoniae*.

The FQRSP isolates belonged to 2 different serotypes and 3 different PFGE types (Figure 1). All belonged to a single ST (ST156). The 3 FQISP isolates from children belonged to serotype 19F. All belonged to a single clone that was different from the adult clone.

### Mechanism of Resistance

We sequenced 7 isolates representing each PFGE type and resistance profile (Table 1, Appendix). Four isolates from adults (nos. 109, 182, 129, 116) had identical mutations previously reported in quinolone resistance: S81F in *gyrA* and S79Y in *parC*. In addition, we observed 3 silent mutations in *gyrB* and 1 silent mutation in *parC*. The silent mutation in *parC* was common to all isolates, including those from the 2 children and the fluoroquinolone-susceptible reference isolate. The 2 isolates from children (nos. 177, 190) had mutations in *parE*; 1 silent mutation and an additional I460V mutation. Isolate no. 200, a susceptible reference, also had an I460V mutation. This mutation is not reported to yield fluoroquinolone resistance. Isolate no. 177, which had a higher degree of resistance to ciprofloxacin and levofloxacin than did isolate no. 190, had 2 additional mutations, E474K and A326V.

**Table 1:** Characterization of *Streptococcus pneumoniae* and quinolone resistance–determining region sequences of type II topoisomerase enzymes GyrA, GyrB, and ParC in a post–acute care facility, Israel, 2006–2011*

Isolate	Serotype	PFGE type	MLST	GyrA		GyrB		ParC		ParE		Etest†			
Missense mutation	Silent mutation	Missense mutation	Silent mutation	Missense mutation	Silent mutation	Missense mutation	Silent mutation	mxf	ofx	cip	Lev
109	19F	D	ST156 Spain 9V-3	**S81F**	–	–	V381, G384, L386	**S79Y**	Q41	–	–	6, R	>32, R	>32, R	>32, R
116	19F	E	ST156 Spain 9V-3	**S81F**	–	–	V381, G384, L386	**S79Y**	Q41	–	–	6, R	>32, R	>32, R	>32, R
129	23F	A2	ST156 Spain 9V-3	**S81F**	–	–	V381, G384, L386	**S79Y**	Q41	–	–	6, R	>32, R	>32, R	>32, R
182	23F	A	ST156 Spain 9V-3	**S81F**	–	–	V381, G384, L386	**S79Y**	Q41	–	–	6, R	>32, R	>32, R	>32, R
190	19F	C	ST81 Spain 23F-1	–	–	–	N461, A472	*K137N*	Q41, G128	I460V	I476	0.19, S	4, I	1.5, S	1, S
177	19F	C	ND	–	–	–	V381, G384, L386	*K137N*	Q41, G128	A326V, **E474K** *I460V*	I476	0.19, S	4, I	6, I	1.5, I
200	15B‡	H	ND	–	–	–	–	–	Q41	*I460V*	–	0.125, S	2, S	1, S	0.75, S

*PFGE, pulsed-field gel electrophoresis; MLST, multilocus sequence typing; ND, not determined; I, intermediate; R, resistance; –, no mutations detected. Boldface font indicates mutations from the literature that conferred fluoroquinolone resistance; underline indicates mutation not recorded in literature; italics font indicates mutation not contributing to fluoroquinolone resistance; regular font indicates silent mutations, all are transition: V381 (gtA-gtG), G384 (ggA-ggG), L386 (ttG-ttA), N461 (aaC aaT), A472 (gcT-gcC), Q41 (caA-caG), G128 (ggC-ggT), I476 (atC-atT)  †Performed on brain-hHeart + blood agar plates and plates were incubated at 35°C CO_2_. ‡15B is a susceptible reference strain.

### Interventions

A total of 197 (83%) eligible patients received PPV23; the remaining 41 patients refused. Seventeen (93%) eligible patients received PCV7.

During 2009, the mean fluoroquinolone DDD in the facility was 36.1. After implementing fluoroquinolone restriction, the mean DDD decreased to 16.7 during 2010 but then increased again to 29.3 during 2011. Total antimicrobial drug use did not change during the entire follow up (DDD was 196, 182, and 208 during 2009, 2010, and 2011, respectively).

### Effect of the Interventions

**Figure 2:**
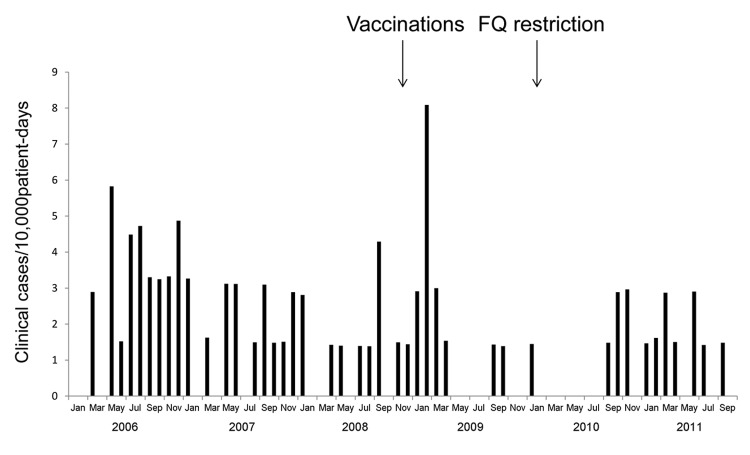
Clinical isolates of FQ-resistant *Streptococcus pneumoniae* in a post–acute care facility, Israel, 2006–2011. FQ, fluoroquinolone.

During the baseline period, the rate of new clinical cases decreased (−0.1021) (Table 2, Figure 2). After implementation of mandatory vaccination, an additional decrease in incidence (−0.4675). However, after the restriction began on use of antimicrobial drugs, incidence again increased (0.5489).

**Table 2:** Trends in the incidence of fluoroquinolone-resistant *Streptococcus pneumonia*e in a post–acute care facility, Israel, 2006–2011

Period	Trend coefficient (SE)	p value
Preintervention	–0.1021 (0.0348)	0.0065
First intervention	–0.4675 (0.1969)	0.0208
Second intervention	0.5489 (0.2071)	0.0102

In the second and third surveys, 154 (90%) of 172 and 165 (97%) of 171 eligible patients were included, respectively. The prevalence of FQRSP decreased from the initial survey to the second survey. Prevalence increased in the third survey (Table 3).

**Table 3:** Results of 3 point-prevalence surveys about *Streptococcus pneumoniae* conducted among residents in a post–acute care facility, Israel, 2006–2011*

Survey and age group, y	No. patients screened	FQRSP			FQISP			FQ-susceptible *S. pneumonia*e	
		No. (%)	Serotype (no. isolates)		No. (%)	Serotype (no. isolates)		No. (%)	Serotype (no. isolates)
First									
<18	15	0	–		2 (13)	15A (1), 19F (1)		2 (13)	19F (2)
>18	69	10 (15)	19F (6), 23F (4)		0	_		6 (8)	15A (1), 15B (2), 23F(1), 6A (2)
Second									
<18	24	0	–		0	_		1 (4)	15A (1)
>18	130	12 (9)	23F (8), 19F (3); negative (1)		0	_		2 (1)	17F (1), 6A (1)
Third									
<18	25	0	–		2 (8)	17F (1), 19F (1)		4 (16)	15A (3), 17F (1)
>18	140	19 (13)	23F (12), 19F (7)		0	_		5 (3.6)	17F (4), negative (1)

*FQRSP, fluoroquinolone-resistant *S. pneumoniae*; FQISP, fluoroquinolone intermediate *S. pneumoniae*; FQ, fluoroquinolone; –, no isolates.

## Discussion

The ability of *S. pneumoniae* to cause outbreaks in long-term care facilities has been reported (*2*,*12*,*13*). Most reports have described invasive infections over a few weeks that involved on average 10–20 patients (*2*,*12*). Also well documented is the ability of multiresistance serotypes to spread internationally and to become predominant clones in multiple geographic areas (*14*–*16*). However, outbreaks of FQRSP have rarely been reported (*4*,*5*). This study describes prolonged transmission of FQRSP in a post–acute care hospital during at least 5 years despite implementation of mandatory vaccination and fluoroquinolone restriction. The prolonged endemicity demonstrates the potential for FQRSP strains to persist within an institution for several years; undergo capsular switch; and in the process, acquire new resistance mutations to multiple antimicrobial drugs.

Most health care–associated *S. pneumoniae* infections are reported from long-term care facilities, and residence in a long-term care facility is an independent risk factor (*2*,*3*,*17*,*18*). In the study reported here, despite the persistence of FQRSP in the facility, we did not notice spread of FQRSP to other settings or health care facilities. During the 5-year follow-up, no FQRSP outbreaks were reported to the National Center for Infection Control from elsewhere in the country, suggesting a high fitness cost of the fluoroquinolone resistance. Despite the prolonged presence of FQRSP clones in the facility, most colonization did not progress to invasive disease. Indeed, the dominant serotypes, 19F and 23F, are not typically associated with invasive disease in adults (*19*). However, reduced virulence as a result of antimicrobial resistance cannot be ruled out. It was previously suggested that penicillin resistance is associated with decreased virulence (*14*,*15*,*20*). However, to the best of our knowledge, no clinical evidence of reduced virulence in FQRSP is available.

Despite the recommendation for PPV23 administration to persons at high risk for pneumococcal pneumonia (*21*), longstanding controversy exists over its efficacy in preventing noninvasive disease (*22*). A recent randomized controlled study demonstrated PPV23 efficacy in preventing pneumococcal pneumonia and reducing associated death in nursing home residents (*23*). Although most successful interventions in long-term care facilities included use of vaccinations (*12*,*24*,*25*), evidence is limited for effectiveness of PPV23 against pneumococcal pneumonia or nasopharyngeal colonization. Furthermore, PPV23 is not thought to reduce carriage and thus cannot be an effective tool to reduce transmission. Preliminary studies suggest that pneumococcal conjugate vaccines induce higher levels of immunity among adults than does PPV23 (*26*). However, further studies are needed to assess whether pneumococcal conjugate vaccines will be more effective in pneumonia prevention among the elderly.

In a recent review, interventions implemented in 28 cluster reports included vaccination, chemoprophylaxis, and infection control measures (*27*). Most studies have reported successful interventions. However, in most cases, outbreak duration and follow-up both were short (median 3 months). In the current study, after implementing mandatory vaccination, we observed an initial decrease in the incidence of FQRSP. However, a decreasing trend was demonstrated even before the intervention. During the prolonged follow-up, we noticed continuous spread of the resistant clones in the facility. Chemoprophylaxis was used in most reported interventions (*27*). However, this strategy might be associated with selection of new resistant strains or mechanisms. Specifically, FQRSP was detected in a long-term care facility, after a short chemoprophylaxis course with combination therapy (*5*). Furthermore, in the present outbreak, because of diversity of the antimicrobial resistance and multiresistance phenotypes, any chosen antimicrobial drug would have further selected and promoted the already prevalent resistant strains, explaining its prolonged persistence. Restriction of fluoroquinolones did not result in a sustained decrease in the incidence of FQRSP. Prior fluoroquinolone treatment was not common in the study population reported here, but the overall use of antimicrobial drugs was not reduced in the studied facility even after the interventions began. We are assessing the effect of antimicrobial drug stewardship and improved compliance with standard precautions on the control of the spread of FQRSP in the facility.

The current FQRSP clone (ST156) comprised 2 different serotypes, which suggests that capsular switch occurred somewhere after introduction of the clone to the facility. The capacity of pneumococci for transformation of capsular type has been described in several studies (*28*,*29*), but in the current study, the location of the event can be assumed with a high degree of certainty. Capsular switch events have been defined as 2 isolates identified by MLST as being closely related but expressing different serotypes. Acquisition of a new capsule may have provided an advantage against the host immune system.

During the past few years, FQRSP has been reported from several countries, although the prevalence remains low (*16*,*30*,*31*). Fluoroquinolone nonsusceptibility among pneumococci results mainly from point mutations in the QRDR topoisomerase genes (*32*). In the current study, identical mutations were found in different serotypes among the strains in adults. This finding suggests that the mutation occurred before capsular switch. Different mutations occurred among the children’s strains and were associated with intermediate resistance. The difference between children and adults might be due to low rates of fluoroquinlone use among children and the relative separation of the children from adults in the family.

Our study has several limitations. First, because microbiological data were not available before January 2006, we cannot determine when the resistant clone was introduced into the facility. Second, clinical isolates for 2006–2008 were not available for analysis. Therefore, we performed molecular characterization only for strains found in the point-prevalence survey. Consequently, we cannot determine the dynamics of resistance development in the facility. Third, we conducted point-prevalence surveys among a sample of hospitalized patients and not among all hospitalized patients. Because we used a convenience sample, selection bias, although unlikely, cannot be categorically excluded. We assessed the effect of both vaccination and fluoroquinolone restriction. However, because the second phase comprised 2 ongoing interventions, we cannot assess separately the effect of each intervention. Finally, we did not conduct a case–control study and therefore risk factors for FQRSP acquisition cannot be assessed.

The persistent transmission of FQRSP during a 5-year period underscores the importance of long-term care facilities as potential reservoir of multidrug resistant particularly FQRSP. Further work is needed to identify optimal strategies to prevent the emergence and spread of resistant pneumococcal strains in long-term care facilities, including potential use of pneumococcal conjugate vaccines, antimicrobial stewardship, and infection control interventions to interrupt transmission.
